# Does Background Matter? A Comparative Characterization of Mouse Models of Autosomal Retinitis Pigmentosa rd1 and Pde6b-KO

**DOI:** 10.3390/ijms242417180

**Published:** 2023-12-06

**Authors:** Angelina V. Chirinskaite, Alexander Yu. Rotov, Mariia E. Ermolaeva, Lyubov A. Tkachenko, Anastasia N. Vaganova, Lavrentii G. Danilov, Ksenia N. Fedoseeva, Nicolay A. Kostin, Julia V. Sopova, Michael L. Firsov, Elena I. Leonova

**Affiliations:** 1 Center of Transgenesis and Genome Editing, St. Petersburg State University, Universitetskaja Emb., 7/9, 199034 St. Petersburg, Russiay.sopova@spbu.ru (J.V.S.); 2 Laboratory of Evolution of Sense Organs, Sechenov Institute of Evolutionary Physiology and Biochemistry, Russian Academy of Sciences, Thorez Ave., 44, 194223 St. Petersburg, Russiadirector@iephb.ru (M.L.F.); 3 Department of Cytology and Histology, St. Petersburg State University, Universitetskaja Emb., 7/9, 199034 St. Petersburg, Russia; 4 Institute of Translational Biomedicine, St. Petersburg State University, Universitetskaja Emb., 7/9, 199034 St. Petersburg, Russia; 5 Department of Genetics and biotechnology, St. Petersburg State University, Universitetskaja Emb., 7/9, 199034 St. Petersburg, Russia; 6 Resource Center “Molecular and Cell Technologies”, St. Petersburg State University, Universitetskaja Emb., 7/9, 199034 St. Petersburg, Russia; 7 Laboratory of Amyloid Biology, St. Petersburg State University, Universitetskaja Emb., 7/9, 199034 St. Petersburg, Russia

**Keywords:** retinitis pigmentosa, *pde6b*, β-subunit of phosphodiesterase, electroretinography, Pde6b-KO

## Abstract

Many retinal degenerative diseases result in vision impairment or permanent blindness due to photoreceptor loss or dysfunction. It has been observed that Pde6b^rd1^ mice (rd1), which carry a spontaneous nonsense mutation in the *pde6b* gene, have a strong phenotypic similarity to patients suffering from autosomal recessive retinitis pigmentosa. In this study, we present a novel mouse model of retinitis pigmentosa generated through *pde6b* gene knockout using CRISPR/Cas9 technology. We compare this Pde6b-KO mouse model to the rd1 mouse model to gain insights into the progression of retinal degeneration. The functional assessment of the mouse retina and the tracking of degeneration dynamics were performed using electrophysiological methods, while retinal morphology was analyzed through histology techniques. Interestingly, the Pde6b-KO mouse model demonstrated a higher amplitude of photoresponse than the rd1 model of the same age. At postnatal day 12, the thickness of the photoreceptor layer in both mouse models did not significantly differ from that of control animals; however, by day 15, a substantial reduction was observed. Notably, the decline in the number of photoreceptors in the rd1 model occurred at a significantly faster rate. These findings suggest that the C3H background may play a significant role in the early stages of retinal degeneration.

## 1. Introduction

Retinitis pigmentosa (RP) is a group of hereditary diseases that lead to visual impairment or permanent blindness due to the loss or dysfunction of photoreceptors and the retinal pigment epithelium. RP is characterized by pronounced genetic and clinical heterogeneity; therefore, the clinical age of the onset of symptoms varies greatly and ranges from childhood to adulthood [1]. To date, autosomal recessive RP has been associated with mutations in at least 74 genes (RetNet: https://sph.uth.edu; accessed 5 July 2023). Recessive biallelic mutations in the *pde6b* gene are a common cause of autosomal recessive RP in both humans and animals [2,3]. According to the Ensembl genome browser database, more than a thousand SNPs in the *pde6b* gene have been found in humans, leading to the development of RP. Since the mouse *pde6b* gene has an orthologous intron–exon relationship comparable to that of the human *pde6b* gene [4], the common phenotypic characteristics of Pde6b inactivation appear, which include the acute degeneration of photoreceptors and their nuclei in the outer nuclear layer, damage to retinal vessels, pigment spots on the fundus, and a decrease in electroretinogram (ERG) amplitudes [5]. The *pde6b* gene encodes the catalytic β-subunit of cyclic guanidine monophosphate (cGMP) phosphodiesterase (Pde6b) expressed specifically in rods’ outer segments. Since Pde6b plays a key role in the conversion of light into electrical response of rod photoreceptor cells, mutations in the *pde6b* gene result in the permanent opening of cGMP-gated cation channels in the photoreceptor membrane, allowing excess extracellular calcium ions to enter these cells, ultimately leading to cell death through apoptosis.

Currently, there are two in vivo mouse models with spontaneous mutations in the *pde6b* gene that are most studied: rd1 (Pde6b^rd1^) and rd10 (Pde6b^rd10^) [3]. Both models exhibit different phenotypes (allelic heterogeneity). The rd1 phenotype is associated with two changes in the *pde6b* locus of chromosome 5: murine leukemia virus insertion (Xmv-28) in reverse orientation (3′ to 5′) in intron 1 and nonsense mutation (p.Y347X, c.C1041A) in exon 7 of the Pde6β subunit. The rd10 phenotype carries a missense mutation (p.R560C, c.C1678T) in exon 13 of the *pde6b* gene and is another model of recessive retinal degeneration. Unlike rd1, in which Pde6b protein production and activity are not detected, rd10 mice are characterized by reduced Pde6b activity and relatively slower onset of retinal degeneration, starting at 4 weeks [6,7]. Residual Pde6b activity in the rd10 retina may prevent the toxic increase in cGMP early in mouse life, thus delaying the onset of photoreceptor cell death and widening the window for therapeutic interventions.

In this work, we established a new mouse model of RP disease with the knockout for the *pde6b* gene. In contrast to the rd1 mouse line, in the *pde6b* gene of our model, a frameshift occurred in exon 1, and there was no insertion of the mouse leukemia virus.

## 2. Results

### 2.1. The Development and Biochemical Characterization of the Pde6b Knockout Mouse Model in Comparison with the rd1 Mouse Model

**Figure 1:**
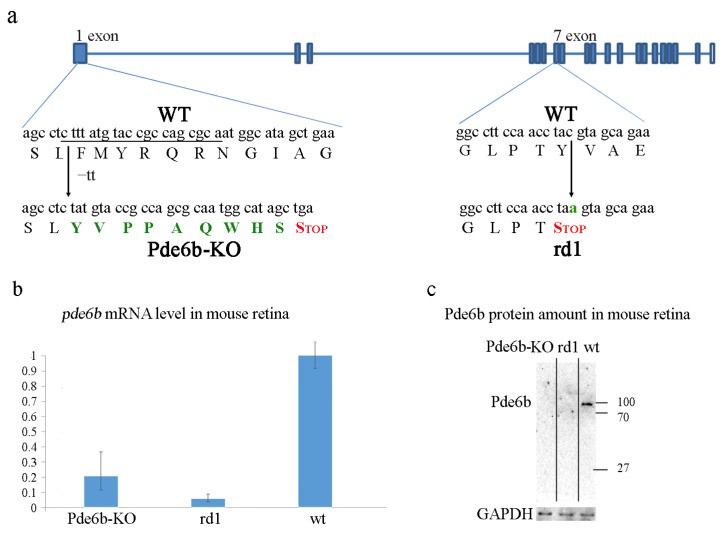
The mutations in the *pde6b* gene in both rd1 and Pde6b-KO mice lead to a decrease in corresponding mRNA levels and a complete absence of the mutant proteins: (**a**) the positions of mutations in rd1 and Pde6b-KO mice; the sequence of sgRNA spacer is underlined, and amino acid substitution and nucleotide substitutions are shown in green; (**b**) the level of *pde6b* mRNA in retinas of wt, rd1, and Pde6b-KO mice on the 9th day; (**c**) the amount of Pde6b protein in mouse retina; the size of Pde6b wt protein is 99 kDa, and the size of Pde6b^rd1^ and Pde6bKO hypothetical proteins is 40 and 11 kDa, respectively.

As we sought to establish a new knockout mouse line lacking the Pde6b protein, we designed sgRNA targeting the first exon of the corresponding gene. A combination of sgRNA and the Cas9 protein was microinjected into mouse zygotes, and 16 F0 mice were genotyped. We selected the F0 mouse model harboring two-nucleotide deletion in the first exon of the *pde6b* gene, leading to a nonsense codon in position 104 (Figure 1).

This mouse model was crossed with a wild-type C57Bl/6 mouse model in order to obtain heterozygous (F1) mice. F1 mice were then crossed to obtain homozygotes and subsequently genotyped via PCR and DNA sequencing analysis.

Furthermore, to eliminate potential off-targets, six backcrosses of homozygotes with wild-type C57Bl/6 mice were performed. Each backcross involved successively crossing homozygous mice with wild-type C57Bl/6 mice and then crossing the resulting heterozygotes with each other to produce homozygotes.

Next, we compared the level of *pde6b* mRNA in the retinas of wild-type, rd1, and Pde6b-KO mice. We found that on the ninth postnatal day, the amount of *pde6b* mRNA in both rd1 and Pde6b-KO mice was drastically decreased (Figure 1). We did not find any detectable amounts of the Pde6b protein both in rd1 and Pde6b-KO retinas on day 12. This corresponds with previously published results on rd1 mice [8].

### 2.2. Electroretinography of Pde6b Knockout Mice in Comparison with rd1 Mice

**Figure 2:**
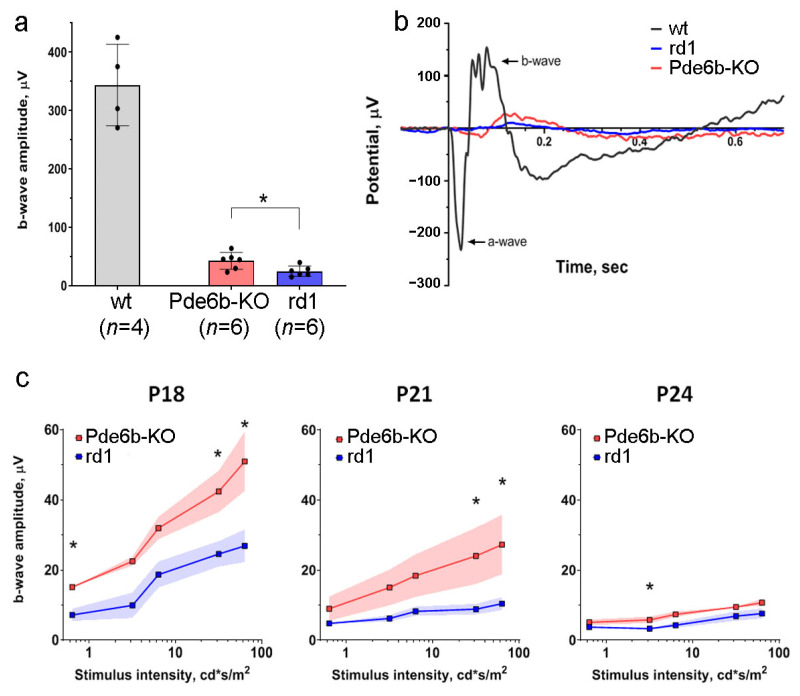
Pde6b-KO and rd1 mice demonstrate different levels of photoresponse amplitude in the electrophysiological analysis: (**a**) comparison between the amplitudes of b-wave ERGs of C57Bl/6 (wild type, wt), Pde6b-KO and rd1 mice on the 18th day of life (*n* = 4–6); the Mann–Whitney test revealed statistically significant differences (* *p* < 0.05); (**b**) comparison of the response shapes in Pde6b-KO and rd1 mice on the 18th day of the postnatal period demonstrated reduced photoresponse in both lines compared to the wild type. The response was measured using a stimulating 10 ms flash with an intensity of 31.6 cd*s/m^2^; (**c**) comparison of b-wave amplitudes in Pde6b-KO and rd1 mice (n = 6) on days 18, 21, and 24 of the postnatal period revealed that the Pde6b-KO photoresponse was higher than that of the rd1 model. The Mann–Whitney test revealed statistically significant differences (* *p* < 0.05).

To assess the functional state of the retina, an electroretinogram (ERG) recording is usually performed in order to measure the electrical response of retinal cells to a light stimulus [9]. The ERG method allows for the calculation of the amplitude and time to the peak of the two primary response components known as the a-wave and the b-wave. The a-wave represents the initial response of photoreceptors to a short flash of light [10], whereas the b-wave reflects the response of underlying retinal neurons, including ON bipolar cells to photoreceptor stimulation. The loss of the a- or b-wave amplitude can be attributed to a number of retinal dystrophies, while “supernormal” waves with increased amplitude have been attributed to cone dystrophy [11]. An analysis of photoresponses demonstrated that the shapes of the ERG on the 18th day of life in Pde6b-KO and rd1 mice were significantly different from that in wild-type animals (Figure 2a,b). Since the rod signaling cascade in rd1 and Pde6b-KO mice lacks a key effector enzyme, they never respond to low-intensity stimuli. Nevertheless, dark-adapted Pde6b-KO mice still exhibited a b-wave response to the bright flash on days 18–21, unlike rd1 mice. However, by the 24th day of life, neither the b-waves nor a-waves were detectable in both strains of mice, indicating a complete loss of visual function (Figure 2c). The responses of both strains on the 27th day were hardly detectable over noise level, and no differences were revealed between them (Figure S1).

### 2.3. Retinal Morphology of Pde6b Knockout Mice in Comparison with rd1 Mice 

In wild-type mice, normal retinal development was observed: the outer nuclear layer (ONL), the inner nuclear layer (INL), and retinal lamination were maintained at all experimental time points (12, 15, 18, and 21 days of postnatal age), and retinal thickness increased, especially in the ONL.

**Figure 3:**
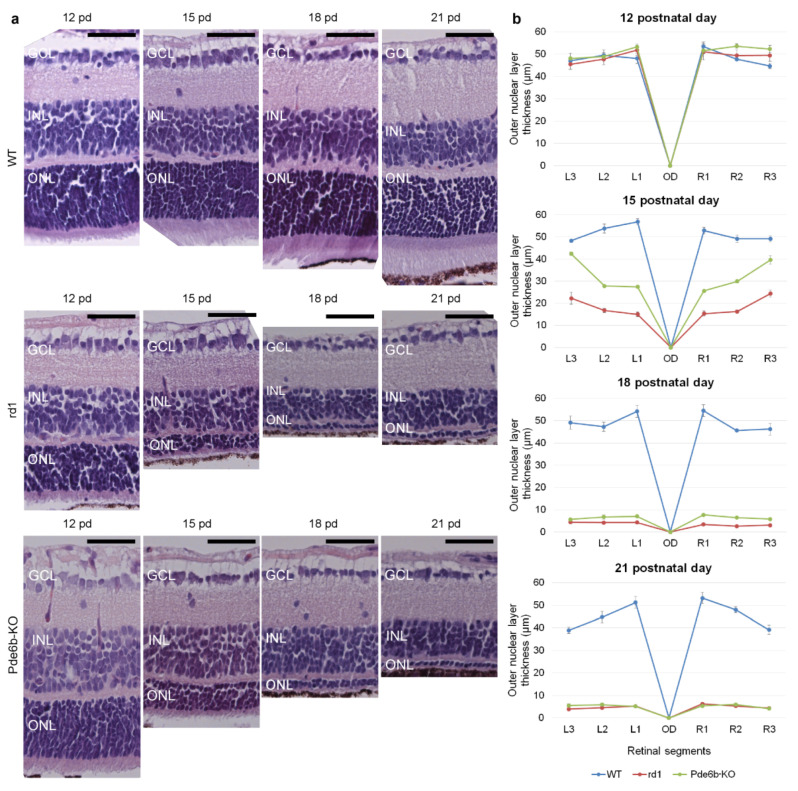
Retinal morphology and morphometry of retinal thickness: (**a**) H&E staining of sections from the central part of the retina of wild-type mice, as well as rd1 and Pde6b-KO mice at 12, 15, 18, and 21 days of postnatal age. ONL, outer nuclear layer (photoreceptor cell bodies); INL, inner nuclear layer; GCL, ganglion cell layer; H&E, hematoxylin and eosin; pd, postnatal day. Scale bars: 50 μm. (**b**) The thickness of the retinal outer nuclear layer of wild-type mice, and rd1 and Pde6b-KO mice at 12, 15, 18, and 21 days of postnatal age. OD, optic disk; L1–L3, R1–R3, measured segments of left and right hemispheres of the eyecup, respectively. Normally, the center is thicker than the periphery, but in models on the 15th day, the thickness of the central parts of the retina sharply decreases.

There was a dramatic decrease in retinal ONL thickness in both models compared to the wild type on the 15th postnatal day. On the 18th postnatal day in both models, there was a decrease in the thickness of the ONL, but in rd1 mice, the ONL consisted of one row of cell nuclei (3 ± 1 μm). In Pde6b-KO mice, the layer thickness was 2–3 cells (10 ± 3 μm). On the 21st day of postnatal age, the ONL was reduced to one row of cell nuclei with the relative preservation of the INL in both rd1 and Pde6b-KO mice (Figure 3a).

The thickness of the ONL gradually decreased in rd1 and Pde6b-KO mice but did not significantly change over time in wild-type mice. On the 15th postnatal day, the ONL was significantly thicker in Pde6b-KO mice at all measured points, compared with those of rd1 mice (*p* < 0.05 for all). By the 21st postnatal day, the ONL thickness in rd1 mice and Pde6b-KO mice was not significantly different (Figure 3b).

### 2.4. Functional Analysis of Genes

In our previous experiments, differences in retina activity and degradation between Pde6b-KO mice and rd1 mice were observed. The Pde6b-KO mouse line had a C57Bl/6 background, and rd1 mice originated from the C3H mouse line, so we hypothesized that this slight difference in retinopathy development could be due to alterations in the genetic background of these mice.

**Figure 4:**
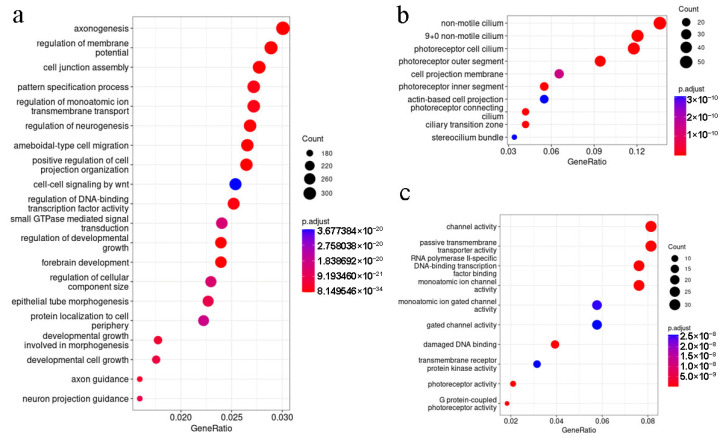
Functional analysis of genes that harbor polymorphisms in C3H mice: (**a**) the dot plot representing the Gene Ontology (GO) biological process terms’ enrichment in the cluster of genes for which nucleotide substitutions in C3H mice were identified (top-20 terms are represented); (**b**) the dot plot representing the Gene Ontology (GO) cell component terms’ enrichment in the cluster of eye development and function-related genes for which nucleotide substitutions in C3H mice were identified (top-10 terms are presented); (**c**) the dot plot representing the Gene Ontology (GO) molecular function terms’ enrichment in the cluster of eye development and function-related genes for which nucleotide substitutions in C3H mice were identified (top-10 terms are presented).

We analyzed 502 genes related to eye development and function that have polymorphisms in C3H mice to assess their role in vision perception with GO enrichment analysis. The genes coding proteins specific to photoreceptors are the most over-represented in this gene set (Figure 4b). Also, the selected gene subcluster is most enriched by the genes described with GO molecular function terms “GO:0015267: Channel activity” and “GO:0008140: Passive transmembrane transporters activity” (Figure 4c).

The analysis demonstrates that the genes that are associated with morphogenesis, especially with neurogenesis, and cell–cell interactions (refer to Figure 4a) are significantly over-represented in the identified cluster of polymorphic genes harboring SNPs in the C3H mouse line compared to the C57Bl/6 mouse model. The significant over-representation of the genes annotated with GO terms associated with retina functioning, including “GO:0060041 retina development in camera-type eye”, “GO:0060042 retina morphogenesis in camera-type eye”, “GO:0031290 retinal ganglion cell axon guidance”, “GO:0003407 neural retina development”, and “GO:0060221 retinal rod cell differentiation”, was also observed.

Additionally, polymorphisms in several genes that could potentially influence retinitis pigmentosa development, including *Rpgrip1*, *Rpgr*, *Gngt1*, *Agtpbp1*, *Grin2a*, *Syngap1*, *Slc24a2*, *Slc24a4*, *Cacnb4*, *Cacna1e*, *Cacna1c*, *Cacna2d4*, *Cacnb2*, *Cib2*, *Pde6c*, *Pde6g*, and *Ppef2*, were further analyzed to predict their functional relevance.

Rpgrip1 has 13 amino acid substitutions (listed in Table S2) and involves the deletions of 2 amino acid residues in positions 971 and 972. The significance of amino acid substitutions was estimated using web tools SIFT [12], PolyPhen2 [13], and PhD-SNP [14]. All substitutions were identified as “neutral” with PhD-SNP and “tolerated” with SIFT. Only with PolyPhen2 were we able to characterize several polymorphisms in the gene *Rpgrip1* as possibly damaging or probably damaging mutations (refer to Table S2). Taking into account that other tools did not reveal any damaging effect of these mutations, the results obtained with PolyPhen2 should be considered with caution, as this tool is characterized by a low specificity rate, with 15–20% of false-positive results [15,16]. The deletion of two glutamic acid residues was localized on the interdomain spacer but could change the protein charge. We assessed the level of *Rpgrip1* mRNA in rd1 and Pde6b-KO mice and found no statistical difference. The results are presented in Figure S2. One amino acid substitution with a neutral effect was identified in the Cacna2d4 protein.

## 3. Discussion

A number of hereditary retinal diseases such as RP lead to vision loss due to the degeneration of photoreceptor cells. The most extensively studied model of RP is the rd1 mouse model with a nonsense mutation in exon 7 of the *pde6b* gene. The Pde6b-KO mouse model presented in this paper differs from the rd1 model in the localization of a frameshift mutation in the *pde6b* gene. The homozygous Pde6b-KO mouse model demonstrated clinical signs of autosomal recessive retinitis pigmentosa due to a two-nucleotide deletion in the *pde6b* gene, leading to a frameshift and nonsense codon in the 104 position. It is assumed that the main cause of photoreceptors’ death in rd1 and Pde6b-KO mice is the increased intracellular calcium influx through continuously opened cGMP-gated channels [17]. Since the stop codon in the rd1 mutant mouse model is located in exon 7, unlike Pde6b-KO, one would expect that the shortened protein would accumulate. And yet, the results of Western blot analysis showed the absence of protein in both models. The functional state of the retina was monitored via in vivo electroretinogram (ERG) recordings. Histological analysis of eyecup cryosections at different time points of postnatal age was performed in order to track morphological changes. The analysis of the ERG revealed that the amplitude of the photoresponse in the Pde6b-KO mouse model at postnatal day 18 was higher than in the rd1 model. The amplitude of the responses continued to decrease until it became indistinguishable from the background noise at 24–27th day. The morphological analysis revealed that in both mouse models, the thickness of the photoreceptor layer at postnatal day 12 did not significantly differ from that of control animals but significantly reduced by day 15. It appears that the decrease in the number of photoreceptors in the rd1 model occurs considerably faster. This difference might arise from different genetic backgrounds because rd1 mice had a mixed origin (C3H/C57Bl/6), whereas Pde6b-KO was obtained on a pure C57Bl/6 background. Using bioinformatic analysis, we found more than 800,000 SNPs that distinguish the C3H and C57Bl/6 mouse lines, and some of them are associated with the development of RP. These data may indicate that the C3H background significantly contributes to the process of retinal degeneration at the early stages of RP development. Further research is needed to establish the exact cause of this phenomenon.

## 4. Materials and Methods

### 4.1. Production of Pde6b KO Mouse Model

All animal studies were approved by the Ethical Committee in the animal research field at Saint Petersburg State University (No 131-03-5, 11 October 2022). All procedures were conducted strictly in accordance with the Guide for the Care and Use of Laboratory Animals. All surgery was performed under general anesthesia using an intraperitoneal injection of TZ (30 mg/kg; Zoletil, Virbac Lab, Carros, France) and xylazine (6 mg/kg; Interchemie Werken de Adelaar, Venray, The Netherlands).

Standard methods of molecular biology and bioinformatics were used to generate CRISPR guide RNA constructs for the *pde6b* gene. The selection of the spacer sequence for the guide RNA was performed using the CHOPCHOP program “http://chopchop.cbu.uib.no/ (accessed on 3 November 2020)”. In vitro transcription was performed using T7 RNA polymerase (Invitrogen, Carlsbad, CA, USA), and the gRNA sequence is listed in Table S1. RNA isolation and purification were performed using a MEGAclear™ Transcription Clean-Up Kit (Invitrogen, Carlsbad, CA, USA).

To obtain fertilized eggs, sexually mature C57BL/6 female mice (4–5 weeks old) (Pushchino, Moscow, Russia) that had undergone superovulation via an intraperitoneal injection of 7U foal mare serum gonadotropin and 7U chorionic gonadotropin and mated overnight with C57BL/6 males (10–18 weeks old) (Pushchino, Moscow, Russia) were used. Zygotes were collected from the oviducts of C57BL/6 female mice with copulation plugs. Thereafter, zygotes were transferred to an M2 embryo culture medium (Sigma, St. Louis, MO, USA) for microinjection. The protein Cas9 mRNA (20 ng/μL) (Invitrogen, Carlsbad, CA, USA) and sgRNA (40 ng/μL) were mixed and microinjected into the cytoplasm of zygotes using a FemtoJet microinjector (Eppendorf, Hamburg, Germany) with constant flow settings. The protocol for cytoplasmic microinjection has been described in detail by Doe et al. [18].

The injected zygotes were cultured in a KSOM medium (Sigma, St. Louis, MO, USA) at 37 °C under 5% CO_2_ for around one hour. Afterward, approximately 8–12 successfully microinjected embryos were transferred into the oviducts of pseudopregnant CD1 mouse females with copulation plugs. In order to obtain CD1 pseudopregnant females (recipients), CD1 females were mated with vasectomized CD1 male mice the day before injection. On the 21st day after birth, F0 pups were genotyped via PCR followed by DNA sequencing analysis. Genomic DNA was extracted from mice’s ears and analyzed with PCR using the primers listed in Table S1. Primers for amplification and sequencing were selected using the PerlPrimer program “http://perlprimer.sourceforge.net/ (accessed on 2 December 2020)”.

The analysis of sequencing chromatograms was performed using the TIDE “https://tide.deskgen.com/ (accessed on 10 January 2021)” computer program.

### 4.2. Electroretinography

To assess the functional state of the retina, the in vivo electroretinography method was used [19]. This technique enables the measurement of changes in the potential on the cornea that occur as a result of retinal responses to light stimuli. The recordings started from the moment when the pup’s eyes were fully opened (18th postnatal day, see [19]). To prepare the mice for ERG recordings, they were kept in darkness for 12 h prior to the procedure. All animal procedures were conducted under dim red lights to maintain darkness. During the recording, each mouse was anesthetized using a Minor Vet anesthesia apparatus (Zoomed, Moscow, Russia) with a gas mixture of isoflurane (2.5%, Laboratorios Karizoo, Spain) and oxygen. Additionally, the local anesthetic oxybuprocaine (inocaine 0.4% solution, Sentiss Rus, Moscow, Russia) and the pupil dilator tropicamide (midrimax 0.8% solution, Sentiss Russ, Moscow, Russia) were applied to both eyes of the mouse. For ERG recording, silver-embedded thread electrodes (Ocuscience, Henderson, NV, USA) were placed on the cornea of the left eye (stimulated by light) and the cornea of the right eye (kept in darkness under the black cap). The ground electrode was securely attached underneath the animal’s skin on the back.

The light stimulation system was equipped with a light-emitting diode that emitted light at a peak wavelength of λmax = 525 nm. The intensity of the light was controlled with a photodiode current and a set of gray neutral-density filters. The intensity of the LED was calibrated with an OPT-301 optosensor (Burr-Brown, Tucson, AZ, USA). Stimuli were presented as brief (10–50 ms) flashes in darkness. Since both rd1 and Pde6b-KO mice lacked rod-derived ERG components, only bright flashes (0.6 cd*s/m^2^ and higher) were able to elicit a response. Signals from electrodes were passed through an amplifier (Model 3000, A-M Systems, Sequim, WA, USA) with a 3 kHz low-frequency analog filter and were recorded at a sampling frequency of 1 ms/point. For data acquisition and controlling the timing, duration, and intensity of the light stimulus during the experiments, the custom-written LabView 7.1 software (National Instruments, Austin, TX, USA) was employed. After baseline correction, the amplitudes of ERG b-waves were calculated and analyzed. All data are presented as the mean ± SEM. A Mann–Whitney U test with a *p*-value < 0.05 was used for statistical analysis.

### 4.3. Histology

Retinal morphology was compared between rd1 (3 males, 3 eyecups), Pde6b-KO (3 males, 3 eyecups), and age-matched wild-type mice (3 males, 3 eyecups) at each time point. At 12, 15, 18, and 21 days of postnatal age, the eyes were enucleated after mice were sacrificed via cervical dislocation. The enucleated whole eyes were prefixed for 5 min in 4% buffered paraformaldehyde, after which the corneas were cut out, and the lens and vitreous body were removed. The remaining eyecups were further fixed for at least 48 h in the same fixative at 4 °C.

Samples were then embedded in paraffin wax for sections (Histomix, Bio-Optica, Saint-Petersburg, Russia). Vertical sections of 6 μm thickness were cut, passing through or close to the optic disc. Specimens were stained using the standard hematoxylin and eosin (H&E) method and were captured using a light microscope fitted with a digital camera (Leica DM 6000, Leica Biosystems Nussloch GmbH, Nussloch, Germany).

For measuring the thickness of the outer nuclear layer (ONL), each hemisphere of the eyecup was divided into 3 equal segments from the optic nerve. At each segment, three independent measurements were performed and averaged. Measurements excluded the first 100 μm from the optic nerve due to the thinness of the retina at this location. At least three sections from each eye were counted, and the averages were used to represent the eye.

The morphometry of retinal layers was determined on the captured digital images of sections using a FIJI v1.53 (Fiji, RRID:SciRes_000137, SCR_002285) image processing package. All data are presented as the mean ± SEM. A Mann–Whitney U test with a *p*-value <0.05 was used for statistical analysis.

### 4.4. Total RNA Extraction and RT-PCR

At the 9th day of postnatal age, the eyes were enucleated under general anesthesia using an intraperitoneal injection of TZ (30 mg/kg; Zoletil, Virbac Lab, Carros, France) and xylazine (6 mg/kg; Interchemie Werken de Adelaar, Venray, The Netherlands). The corneas were cut out, and the lens and vitreous body were removed. The remaining eyecups were treated with RNAprotect reagent (Promega, Madison, WI, USA), and the total RNA was extracted with an RNeasy Mini Kit (Qiagen, Hilden, Germany) according to the manufacturer’s instructions. The RNA concentration and quality were measured after gel electrophoresis using the ChemiDoc XRS+ Gel Imaging System (Bio-Rad, Hercules, CA, USA) and Image Lab 6.0.1. software (Bio-Rad, Hercules, CA, USA). cDNA was synthesized using an iScript cDNA Synthesis Kit (Bio-Rad, Hercules, CA, USA), and the primers for RT-PCR are listed in Table S1. The 2^−∆∆Ct^ method was used to calculate the difference in the relative expression levels of the target genes normalized on the housekeeping gene *GAPDH*.

### 4.5. Protein Extraction and Western Blot Hybridization

On day 12 of postnatal age, the eyes were enucleated under general anesthesia using an intraperitoneal injection of TZ (30 mg/kg; Zoletil, Virbac Lab, Carros, France) and xylazine (6 mg/kg; Interchemie Werken de Adelaar, Venray, The Netherlands). The corneas were cut out, and the lens and vitreous body were removed. The remaining eyecups were homogenized using 0.5 µm glass beads (Sigma, St. Louis, MO, USA) in lysis buffer (25 mM Tris-HCl, pH 7.4, 100 mM NaCl, 1 mM dithiothreitol, 10 mM EDTA, and 2 mM PMSF) and a Complete™ protease inhibitor mixture (Roche Applied Science, Penzberg, Germany). The total protein concentration was measured using a Qubit 2.0 fluorometer and a protein assay kit (Invitrogen, Carlsbad, CA, USA), 35 μg of the total protein extracts was used per lane. The Pde6b protein was detected with the primary Anti-PDE6B Polyclonal antibody PA1-722 (Invitrogen, Carlsbad, CA, USA) and secondary HRP-conjugated Goat Anti-Rabbit IgG H&L ab205718 (Abcam, Cambridge, UK). The GAPDH protein was detected with the primary Anti-GAPDH polyclonal antibody AF7021 (Affinity Biosciences, Zhenjiang, China) and secondary HRP-conjugated Goat Anti-Rabbit IgG H&L ab205718 (Abcam, Cambridge, UK). Chemiluminescent detection was performed using an Amersham ECL Prime Western Blotting Detection Reagent (GE Healthcare, Chicago, IL, USA).

### 4.6. The Identification and Description of Genetic Polymorphisms 

The C3H/HeJ mouse genome provided in the NCBI database (NCBI GenBankID GCA_921997125.2) was compared with the reference C57BL/6 genome (NCBI RefSeqID GCF_000001635.27) using the minimap2 tool (version 2.26) [20] with default settings. Post-processing was performed using a custom Python script.

Protein sequences were downloaded from the Ensembl database [21]. The identified amino acid substitutions’ effects were predicted using the bioinformatic web tools SIFT (Sorting Intolerant From Tolerant, available at https://sift.bii.a-star.edu.sg/ (accessed on 11 July 2023)) [12], PolyPhen2 (Polymorphism Phenotyping v-2, available at http://genetics.bwh.harvard.edu/pph2/index.shtml (accessed on 11 July 2023)) [13], and PhD-SNP (available at https://snps.biofold.org/phd-snp/phd-snp.html (accessed on 11 July 2023)) [14].

### 4.7. Function and Enrichment Analysis

Gene Ontology (GO) term enrichment analysis (i.e., the identification of GO terms [22] that are significantly enriched by the genes of the selected set) was performed in the gene cluster, which included the genes that harbor nucleotide substitutions in C3H mice compared to the C57Bl/6 mice genome. The enriched terms were identified and visualized using the clusterProfiler Bioconductor package [23]. Biologic process (BP) ontology was used [22].

Then, the GO enrichment analysis was performed in the same way in the cluster, which includes the selected genes associated with retina development and functioning. Cell component (CC) and molecular function (MF) ontologies were used [22].

### 4.8. Functional Analysis of Genes Harboring Nucleotide Polymorphisms in C3H

The alignment of C3H and C57Bl/6 mouse genomes led to the identification of 221,946 nucleotide substitutions in 15,597 genes in C3H compared to the reference C57Bl/6 genome sequence. To explain the specific biological function of the genes in which polymorphisms were identified, we performed the biological process GO terms’ enrichment analysis.

## 5. Patents

The patent for the proposed method for the generation and application of a mouse model with *pde6b* gene knockout was obtained with Patent Number RU 2 791 687 C1.

## Data Availability

All data are available upon reasonable request.

## Supplementary Materials

The supporting information can be downloaded at: https://www.mdpi.com/article/10.3390/ijms242417180/s1.

## References

[1] Daiger S.P., Sullivan L.S., Bowne S.J. (2013). Genes and Mutations Causing Retinitis Pigmentosa. Clin. Genet..

[2] Aziz N., Ullah M., Rashid A., Hussain Z., Shah K., Awan A., Khan M., Ullah I., Rehman A.U. (2023). A Novel Homozygous Missense Substitution p.Thr313Ile in the PDE6B Gene Underlies Autosomal Recessive Retinitis Pigmentosa in a Consanguineous Pakistani Family. BMC Ophthalmol..

[3] Yeo J.H., Jung B.K., Lee H., Baek I.-J., Sung Y.H., Shin H.-S., Kim H.K., Seo K.Y., Lee J.Y. (2019). Development of a Pde6b Gene Knockout Rat Model for Studies of Degenerative Retinal Diseases. Invest. Ophthalmol. Vis. Sci..

[4] Wu W.-H., Tsai Y.-T., Justus S., Lee T.-T., Zhang L., Lin C.-S., Bassuk A.G., Mahajan V.B., Tsang S.H. (2016). CRISPR Repair Reveals Causative Mutation in a Preclinical Model of Retinitis Pigmentosa. Mol. Ther..

[5] Collin G.B., Gogna N., Chang B., Damkham N., Pinkney J., Hyde L.F., Stone L., Naggert J.K., Nishina P.M., Krebs M.P. (2020). Mouse Models of Inherited Retinal Degeneration with Photoreceptor Cell Loss. Cells.

[6] Han J., Dinculescu A., Dai X., Du W., Smith W.C., Pang J. (2013). Review: The History and Role of Naturally Occurring Mouse Models with Pde6b Mutations. Mol. Vis..

[7] Wang T., Reingruber J., Woodruff M.L., Majumder A., Camarena A., Artemyev N.O., Fain G.L., Chen J. (2018). The PDE6 Mutation in the Rd10 Retinal Degeneration Mouse Model Causes Protein Mislocalization and Instability and Promotes Cell Death through Increased Ion Influx. J. Biol. Chem..

[8] van Wyk M., Schneider S., Kleinlogel S. (2015). Variable Phenotypic Expressivity in Inbred Retinal Degeneration Mouse Lines: A Comparative Study of C3H/HeOu and FVB/N Rd1 Mice. Mol. Vis..

[9] Leinonen H., Tanila H. (2018). Vision in Laboratory Rodents—Tools to Measure It and Implications for Behavioral Research. Behav. Brain Res..

[10] Vollrath D., Yasumura D., Benchorin G., Matthes M.T., Feng W., Nguyen N.M., Sedano C.D., Calton M.A., LaVail M.M. (2015). Tyro3 Modulates Mertk-Associated Retinal Degeneration. PLoS Genet..

[11] Benchorin G., Calton M., Beaulieu M., Vollrath D. (2017). Assessment of Murine Retinal Function by Electroretinography. Bio-Protocol.

[12] Lowe D.G. Object Recognition from Local Scale-Invariant Features. Proceedings of the Seventh IEEE International Conference on Computer Vision.

[13] Adzhubei I.A., Schmidt S., Peshkin L., Ramensky V.E., Gerasimova A., Bork P., Kondrashov A.S., Sunyaev S.R. (2010). A Method and Server for Predicting Damaging Missense Mutations. Nat. Methods.

[14] Capriotti E., Fariselli P. (2017). PhD-SNPg: A Webserver and Lightweight Tool for Scoring Single Nucleotide Variants. Nucleic Acids Res..

[15] Flanagan S.E., Patch A.-M., Ellard S. (2010). Using SIFT and PolyPhen to Predict Loss-of-Function and Gain-of-Function Mutations. Genet. Test. Mol. Biomark..

[16] Masica D.L., Sosnay P.R., Cutting G.R., Karchin R. (2012). Phenotype-Optimized Sequence Ensembles Substantially Improve Prediction of Disease-Causing Mutation in Cystic Fibrosis. Hum. Mutat..

[17] Sancho-Pelluz J., Arango-Gonzalez B., Kustermann S., Romero F.J., Van Veen T., Zrenner E., Ekström P., Paquet-Durand F. (2008). Photoreceptor Cell Death Mechanisms in Inherited Retinal Degeneration. Mol. Neurobiol..

[18] Doe B., Brown E., Boroviak K. (2018). Generating CRISPR/Cas9-Derived Mutant Mice by Zygote Cytoplasmic Injection Using an Automatic Microinjector. Methods Protoc..

[19] Goriachenkov A.A., Rotov A.Y., Firsov M.L. (2021). Developmental Dynamics of the Functional State of the Retina in Mice with Inherited Photoreceptor Degeneration. Neurosci. Behav. Physiol..

[20] Li H. (2021). New Strategies to Improve Minimap2 Alignment Accuracy. Bioinformatics.

[21] Cunningham F., Allen J.E., Allen J., Alvarez-Jarreta J., Amode M.R., Armean I.M., Austine-Orimoloye O., Azov A.G., Barnes I., Bennett R. (2022). Ensembl 2022. Nucleic Acids Res..

[22] Ashburner M., Ball C.A., Blake J.A., Botstein D., Butler H., Cherry J.M., Davis A.P., Dolinski K., Dwight S.S., Eppig J.T. (2000). Gene Ontology: Tool for the Unification of Biology. Nat. Genet..

[23] Wu T., Hu E., Xu S., Chen M., Guo P., Dai Z., Feng T., Zhou L., Tang W., Zhan L. (2021). ClusterProfiler 4.0: A Universal Enrichment Tool for Interpreting Omics Data. Innovation.

