# Supplementary material for: Does Background Matter? A Comparative Characterization of Mouse Models of Autosomal Retinitis Pigmentosa rd1 and Pde6b-KO
Source: Int J Mol Sci. 2023 Dec 6;24(24):17180. doi: 10.3390/ijms242417180 (PMC10742838; doi:10.3390/ijms242417180)
Supplement: Supplementary file 1 [file ijms-24-17180-s001.zip › Supplemental figures.pdf]

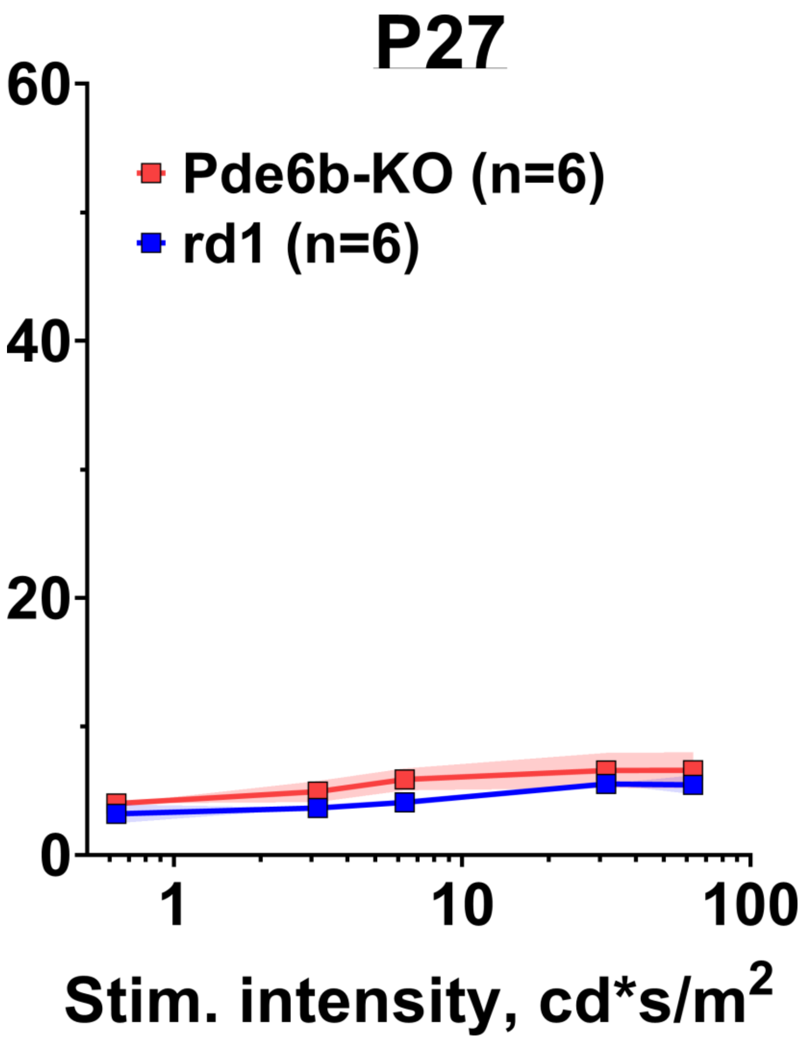

Figure S1. Comparison of b-wave amplitudes in Pde6b-KO and rd1 mice (n=6) on 27th day of the postnatal period. Responses of both strains are hardly detectable over noise level and no differences revealed between them (Mann-Whitney test, $p > 0.05$).

The level of *Rpgrip1* mRNA

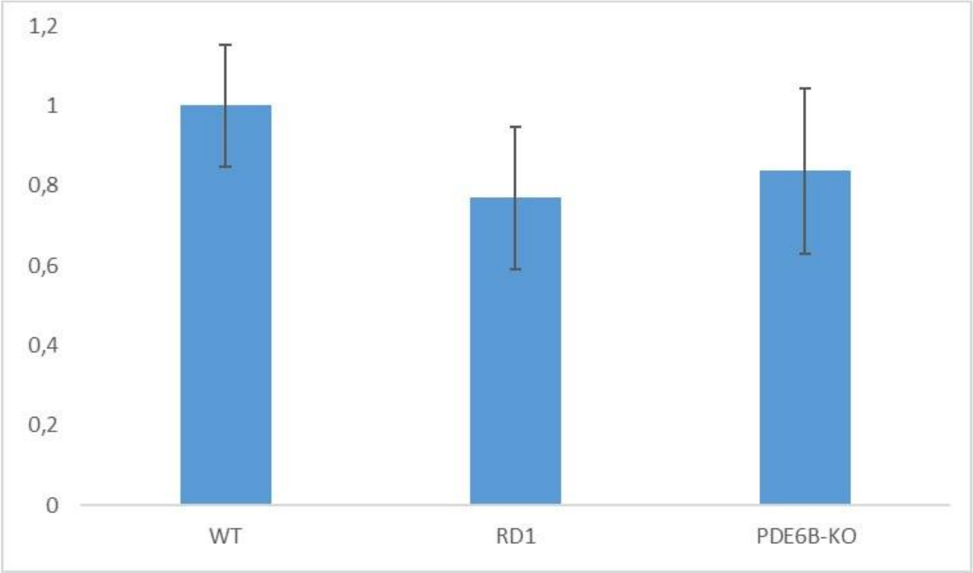

Figure S2. The level of *Rpgrip1* mRNA on the 9th day does not change significantly in rd1 and Pde6-KO mice compared to wt mice.
